# Healthcare workers’ mental health and perception towards vaccination during COVID-19 pandemic in a Pediatric Cancer Hospital

**DOI:** 10.1038/s41598-022-24454-5

**Published:** 2023-01-06

**Authors:** Mai Alalawi, Mohamad Makhlouf, Omnya Hassanain, Ahmed A. Abdelgawad, Mohamed Nagy

**Affiliations:** 1 Department of Pharmaceutical Services, Children’s Cancer Hospital Egypt (57357), Cairo, Egypt; 2 Department of Pharmaceutical Sciences, Fakeeh College for Medical Sciences, Jeddah, Saudi Arabia; 3 Department of Epidemiology and Clinical Research, Children’s Cancer Hospital Egypt (57357), Cairo, Egypt; 4 Department of Psychiatry, Children’s Cancer Hospital Egypt (57357), Cairo, Egypt; 5 Department of Psychiatry, Faculty of Medicine, Ain Shams University, Cairo, Egypt; 6 Personalized Medication Management Unit, Children’s Cancer Hospital Egypt (57357), Cairo, Egypt

**Keywords:** Psychology, Human behaviour

## Abstract

The consistent increase of Coronavirus disease 2019 (COVID-19) cases parallel with the rate of deaths and the controversial response regarding the vaccines caused an increase in the burden of psychological diseases. This study aimed to evaluate the psychological condition of healthcare workers (HCWs) in a pediatric cancer hospital and to identify the knowledge, attitude, and perception (KAP) of HCWs toward COVID-19 vaccination. A cross-sectional observational study was conducted between April to May 2021. A validated, confidential survey was employed to measure the mental health of HCWs and the KAP toward COVID-19 vaccines. The total responses were 395, of which 11.4% physicians, 18.5% pharmacists, and 70.1% were nurses. Sixty-six percent of HCWs had different degrees of anxiety and depression. Nurses significantly accounted for the highest anxiety levels (*P* = 0.003), while the cumulative anxiety score was significantly higher in HCWs who had a positive history of COVID-19 infection (*P* = 0.026). Although 67.6% of HCWs believe that “vaccines are essential for us,”, the vaccination rate was 21.3%. The Factors associated with not receiving the vaccine were younger ages (*P* = 0.014), nurses (*P* = 3.6987 × 10^–7^), negative history of COVID-19 infection (*P* = 0.043) and believing that infections can happen after taking the vaccine (*P* = 1.5833 × 10^–7^). Healthcare organizations must take serious intervention to decrease the mental load on HCWs and facilitate the vaccination process.

## Introduction

Coronavirus disease 2019 (COVID-19) is a respiratory infectious disease that the World Health Organization (WHO) declared it as a pandemic during 2020^1^. The causative virus; severe acute respiratory syndrome coronavirus 2 (SARS-CoV2) infected over two hundred million worldwide and resulted in more than four million deaths until September 2021^1,2^. In Egypt, the cumulative number of the confirmed cases is 294,482, and 16,908 deaths due to COVID-19 in September 2021^2^. The consistent increase of cases parallel with the rate of deaths causes an increase in the workload and the demand for healthcare workers (HCWs). In addition, the extensive media coverage, rumors and fake news, and controversial response regarding the available vaccines put extra pressure on HCWs. This altogether increases the burden of psychological diseases such as anxiety and depression^3–5^.

HCWs are the most exposed category to psychological dysfunctions^6^. Over history, infectious diseases’ outbreaks were associated with emotional distress and anxiety symptoms among HCWs. Moreover, anxiety, depression, and burnout were also reported in HCWs after the outbreaks were over^7,8^. Despite that, most HCWs do not receive training to provide mental health care^9^. As a result, the extreme pressure on HCWs during COVID-19 led them to suffer from psychological and mental problems^5^.

A systematic review was carried out to investigate the psychological impact of COVID-19 in HCWs in different countries. Eleven articles were included in the analysis and identified the prevalence of anxiety ranging from 24.1 to 44.6%. In addition, the prevalence of moderate to severe depression ranged from 12.1% to 50.4%.Severe degrees of symptoms were reported in frontline nurses, younger ages, and female workers^5^. In Egypt, a study conducted in 20 hospitals (502 HCWs) reported that 76.4% had an abnormality of anxiety scale, 67.7% had positive insomnia symptoms, and 77.2% showed symptoms of depression^10^.

The effect of the pandemic on HCWs mental health can vary individually or based on particular circumstances^11^. A cross-sectional study conducted in Egypt in May 2020 (540 HCWs) surveyed HCWs working in quarantine hospitals and found that adverse psychological symptoms were significantly predicted in younger ages HCWs, those who were not ready to work in quarantine hospitals, and HCWs who had insomnia^12^. Working with suspected or confirmed COVID-19 cases has been challenging for HCWs, resulting in negative psychological impact^11^. In addition, supporting oncology patients and maintaining their services during the pandemic is another huge challenge^13^.

Responding to the epidemic, hundreds of vaccines have gone through experimentation registered on ClinicalTrials.gov. Different technologies for the vaccines’ development were used, such as viral vectors vaccines, inactivated vaccines, RNA vaccines, and others. A systematic review that included 13 randomized clinical trials confirmed the efficacy and safety of several COVID-19 vaccines^14^. Several vaccines are now being administered globally. To date, all the data recommends the efficacy of COVID-19 vaccines in providing significant protection against COVID-19 infection. However, the long-term efficacy and safety are not yet established^14^.

There is controversy regarding taking the vaccine or not^15^. In a systematic review that estimated the vaccine acceptance rates in 33 countries reported the lowest acceptance rates in the Middle East, Africa, Russia, and many European countries. In addition, the acceptance of the vaccine among doctors and nurses ranged from 27.7 to 78.1% based on the country. Similarly, in a study conducted in Bangladesh, 60% of the participants said they plan to take the vaccine^15^. The hesitancy to take COVID-19 vaccines negatively affects the global efforts toward controlling the pandemic^16^.

A lower vaccination rate in any country can result in new variants that the current vaccines may fail to protect us. A study that analyzed 15 surveys on vaccine acceptance in lower- and middle-income countries found that the most common reason for vaccines refusal is side effects^17^. However, a national survey conducted among the United States citizens suggested that responders are influenced to take COVID-19 vaccines when they understand the expected benefits rather than the expected side effects^18^.

The degree of pandemic effect on mental status is now a global concern^11^. Moreover, HCWs' decision-making and physiological functioning rely on their psychological status to be assessed and addressed^19^. Few studies have been published that evaluated employees' mental health in working in healthcare facilities in Egypt, and the limited evidence regarding the acceptance of vaccination in the Egyptian population, especially HCWs. Thus, the aim of the present study is to evaluate the psychological condition of HCWs in a pediatric cancer hospital using validated scales and to explore the factors associated with psychological symptoms, such as sociodemographic data and occupation circumstances. Also, it aims to identify the knowledge, attitude, perception (KAP), and acceptance of HCWs toward COVID-19 vaccination.

## Methods

### Study design

A cross-sectional observational study (hospital-based survey) was conducted in Children’s Cancer Hospital 57,357 (CCHE), Cairo, Egypt.

### Population and sample size

The target population was HCWs at CCHE. The inclusion criteria were licensed physicians, pharmacists, and nurses working at CCHE who completed the survey during the time of the data collection, while the exclusion criteria were HCWs who reported a positive history of depression, anxiety, or other previous phycological illness and HCWs who reported receiving medication for a psychological condition. The sample size was calculated using the sample-to-variable ratio method of 15:1^20,21^. For the pilot and the main survey, at least 50 and 375 subjects were recommended, respectively. A minimum number was recommended for each profession as follows: for the pilot study: 8 pharmacists, 5 physicians, and 37 nurses were recommended, while for the main survey: 40 physicians, 59 pharmacists, and 276 nurses.

### Data collection

The survey was distributed between April 2021 to May 2021 to all HCWs in two forms: paper-based or electronic. The duration of data collection was parallel to the arrival of COVID-19 vaccines in the country. An informed consent was written at the beginning of the study that stated the aim of the study, and the obtained anonymous data will be only used for the study. The agreement on the informed consent was accomplished by completing the survey.

### Survey tools

A validated, confidential survey was conducted to obtain the data required for the study. Experts in the field assessed the face and content validity of the survey. A pilot study was conducted first to examine the feasibility of the study that is intended to be used on a larger scale ultimately. The survey consisted of three sections; the first section included demographic data, questions regarding the history of the infection, the history of working in COVID-19 unites, and the history of the psychiatric illness. The second section consisted of two validated scales; generalized anxiety disorder 7-item GAD-7 scale^22^ and patient health questionnaire PHQ-9^23^ to assess anxiety and depression levels, respectively. The third section included general questions assessing the KAP toward COVID-19 vaccines. The questions were in (yes OR no) or multiple choices. The primary outcome was the extent of depression and anxiety. Other outcomes of interest were the factors associated with depression and anxiety and the acceptance of COVID-19 vaccination^15^.

### Statistical analysis

The data were analyzed and graphed using Microsoft Excel® and IBM’s SPSS Statistics package (version 26). Descriptive statistics (frequency, percentages, mean, standard deviation) were performed for socio-demographics and the survey questions’ responses. The Chi-square test was used for categorical variables, while Mann–Whitney U test was used for continuous variables. A *P* value < 0.05 was considered to be statistically significant.

### Ethical approval and consent to participate

The study was approved by the institutional review board in Children’s Cancer Hospital 57,357 (CCHE). The informed consent of participants was gained at the beginning of the survey.

## Results

### Sociodemographic characteristics of the study sample

A total of 415 HCWs participated in the survey. Of which, 20 responses were excluded due to positive history of psychiatric illness and/or receiving medication for a psychiatric condition. Thus, the included number for the analysis was 395 responses, of which 45 (11.4%) were physicians, 73 (18.5%) were pharmacists, and 277 (70.1%) were nurses. The corresponding response rate per profession was 50.36% for nurses, 48.67% for pharmacists, and 45% for physicians. Female participants accounted for 54.94%, and more than half of the participants lay between the age group 25–35 years (55.19%). In addition, approximately one-third had extra credentials after their bachelor’s degree. One hundred eighty-seven (47.3%) of the participants reported a positive history of COVID-19 infection, and 112 (28.4%) worked in a COVID-19 isolation unit. The sociodemographic and basic characteristics of the total included sample are illustrated in Table 1.

**Table 1:** Sociodemographic data and basic characteristics of HCWs working at CCHE who responded to the survey.

Characteristic	N (%) (Total N = 395)
**Age (years)**	
< 25	97 (24.56%)
25–35	218 (55.19%)
36–45	64 (16.20%)
> 45	16 (4.05%)
**Gender**	
Male	178 (45.06%)
Female	217 (54.94%)
**Profession**	
Physicians	45 (11.4%)
Assistant consultant	2
Consultant	12
Fellowship	2
Resident	14
Specialist	15
Pharmacists	73 (18.5%)
Ambulatory care	4
Dispensing pharmacist	16
IV/Admixture pharmacist	10
Ward pharmacist	8
Specialty pharmacist	35
Nurses	277 (70.1%)
Charge nurse	66
Clinical instructor	5
Head nurse	15
Staff nurse	191
**Extra credentials**	
Yes	134 (33.9%)
No	261 (66.1%)
**Years of Practicing the profession**	
< 5 years	170 (43.04%)
5–9.9 years	134 (33.92%)
10–14.9 years	56 (14.18%)
15–19.9 years	21 (5.32%)
≥ 20 years	14 (3.54%)
**History of COVID-19 infection**	
Yes	187 (47.3%)
No	208 (52.7%)
**Endorsed history of working in a COVID-19 Unit**	
Yes	112 (28.4%)
No	283 (71.6%)
Median working hours/day ± SD*	12 ± 4.3 h

*SD = standard deviation.

### Anxiety assessment and associated factors

The anxiety level was estimated using the GAD-7 scale (the total score is 21). A score of (0–4) is considered minimal anxiety, (5–9) mild anxiety, (10–14) moderate anxiety, and (15–21) severe anxiety^22^. A considerable number of HCWs (261, 66.1%) had abnormal anxiety degrees on the scale mild to severe, 148 (37.5%) had mild anxiety, 76 (19.2%) had moderate anxiety, and 37 (9.4%) had severe anxiety. Non-statistical significance was observed when comparing age groups, gender, years of practice, positive history of COVID-19 infection, endorsed a history of working in a COVID-19 unit with the anxiety level and anxiety score. However, among different professions, nurses had the highest levels of anxiety in comparison to physicians and pharmacists, and it was statistically significant (*P* = 0.003) (Table 2). Also, the cumulative anxiety score was significantly higher in HCWs who had a positive history of COVID-19 infection (*P* = 0.026) (Table 3).

**Table 2:** The level of anxiety among HCWs (using GAD-7 scale).

Variable N (% within category)	Minimal anxiety	Mild anxiety	Moderate anxiety	Severe anxiety	*P*-value
Total N = 395	134 (33.9%)	148 (37.5%)	76 (19.2%)	37 (9.4%)	
**Age**					
< 25	40 (41.24%)	27 (27.84%)	17 (17.52%)	13 (13.4%)	0.120
25–35	69 (31.7%)	88 (40.4%)	46 (21%)	15 (6.9%)	
36–45	21 (32.8%)	27 (42.2%)	11 (17.2%)	5 (7.8%)	
> 45	4 (25%)	6 (37.5%)	2 (12.5%)	4 (25%)	
**Gender**					
Male	64 (36%)	69 (38.8%)	29 (16.3%)	16 (8.9%)	0.562
Female	70 (32.3%)	79 (36.4%)	47 (21.7%)	21 (9.6%)	
**Profession**					
Physicians	7 (15.5%)	25 (55.6%)	12 (26.7%)	1 (2.2%)	0.003
Pharmacists	21 (28.8%)	24 (32.9%)	16 (21.9%)	12 (16.4%)	
Nurses	106 (38.3%)	99 (35.7%)	48 (17.3%)	24 (8.7%)	
**Years of practice**					
< 5 years	60 (35.3%)	59 (34.7%)	31 (18.2%)	20 (11.8%)	0.293
5 – 9.9 years	47 (35.1%)	51 (38.1%)	29 (21.6%)	7 (5.2%)	
10 – 14.9 years	12 (21.43%)	27 (48.21%)	12 (21.43%)	5 (8.93%)	
15 – 19.9 years	9 (42.9%)	8 (38.1%)	2 (9.5%)	2 (9.5%)	
≥ 20 years	6 (42.9%)	3 (21.4%)	2 (14.3%)	3 (21.4%)	
**History COVID-19 infection**					
Yes	55 (29.4%)	75 (40.1%)	39 (20.9%)	18 (9.6%)	0.348
No	79 (38%)	73 (35.1%)	37 (17.8%)	19 (9.1%)	
**History of working in a COVID-19 Unit**					
Yes	41 (36.6%)	38 (33.9%)	18 (16.1%)	15 (13.4%)	0.224
No	93 (32.8%)	110 (38.9%)	58 (20.5%)	22 (7.8%)

**Table 3:** The history of COVID-19 infection and anxiety score.

History of COVID-19 infection	Anxiety score Mean ± SD* 95% CI*	P-value
Positive History	7.52 ± 4.85 (6.82–8.22)	**P = 0.026**
Negative History	6.75 ± 4.89 (6.08–7.42)	

*SD = standard deviation, CI = confidence interval for the mean.

Significant values are in bold.

### Depression assessment and associated factors

The level of depression was assessed using the PHQ-9 questionnaire (the total score is 27). The interpretation of the score is as follows: non-minimal (1–4), mild depression (5–9), moderate depression (10–14), moderately severe (15–19), and severe depression (20–27)^23^. Two hundred sixty-one HCWs (66%) had abnormal degrees of depression (mild to severe). Higher levels of depression were observed in HCWs who had a previous history of working in COVID-19 units or had COVID-19 infection. The depression level and score were not significant compared to all the factors Table 4.

**Table 4:** The level of depression among HCWs (using PH-Q9 scale).

Variable N (% within category)	Non-minimal	Mild depression	Moderate depression	Moderately severe depression	Severe depression	*P*-value
Total N = 395	134 (33.9%)	155 (39.2%)	62 (15.7%)	30 (7.6%)	14 (3.5%)	
**Age**						
< 25	31 (32%)	38 (39.2%)	14 (14.4%)	11 (11.3%)	3 (3.1%)	0.903
25–35	74 (33.94%)	89 (40.83%)	32 (14.68%)	16 (7.34%)	7 (3.21%)	
36–45	23 (35.94%)	23 (35.94%)	13 (20.3%)	2 (3.13%)	3 (4.69%)	
> 45	6 (37.5%)	5 (31.25%)	3 (18.75%)	1 (6.25%)	1 (6.25%)	
**Gender**						
Male	62 (34.8%)	71 (39.9%)	29 (16.3%)	10 (5.6%)	6 (3.4%)	0.758
Female	72 (33.2%)	84 (38.7%)	33 (15.2%)	20 (9.2%)	8 (3.7%)	
**Profession**						
Physicians	14 (31.1%)	21 (46.7%)	6 (13.3%)	3 (6.7%)	1 (2.2%)	0.740
Pharmacists	22 (30.14%)	31 (42.46%)	10 (13.7%)	5 (6.85%)	5 (6.85%)	
Nurses	98 (35.4%	103 (37.2%)	46 (16.6%)	22 (7.9%)	8 (2.9%)	
**Years of practice**						
< 5 years	52 (30.6%)	68 (40%)	27 (15.9%)	18 (10.6%)	5 (2.9%)	0.58
5–9.9 years	54 (40.3%)	49 (36.6%)	18 (13.4%)	8 (6%)	5 (3.7%)	
10–14.9 years	13 (23.2%)	25 (44.6%)	13 (23.2%)	3 (5.4%)	2 (3.6%)	
15–19.9 years	9 (42.86%)	9 (42.86%)	2 (9.52%)	0 (0%)	1 (4.76%)	
≥ 20 years	6 (42.9%)	4 (28.6%)	2 (14.3%)	1 (7.1%)	1 (7.1%)	
**History COVID-19 infection**						
Yes	56 (29.9%)	71 (38%)	34 (18.2%)	18 (9.6%)	8 (4.3%)	0.225
No	78 (37.5%)	84 (40.38%)	28 (13.46%)	12 (5.77%)	6 (2.89%)	
**History of working in a COVID-19 Unit**						
Yes	35 (31.25%)	42 (37.5%)	20 (17.85%)	9 (8.04%)	6 (5.36%)	0.662
No	99 (34.98%)	113 (39.93%)	42 (14.84%)	21 (7.42%)	8 (2.83%)	

### Knowledge, attitude, and perception about COVID-19 vaccines

The responses to the KAP assessment section are shown in Table 5. The participants were asked if they knew about the vaccine efficacy and safety; 80% and 83.3% answered yes, respectively. For the knowledge item, the comparison of the responses between professions was statistically significant regarding knowing that the infection can happen after taking the vaccine (*P* = 7.018 × 10^–9^). In addition, 67.6% believe that the vaccines are essential for us, while only 21.3% received at least one dose of a COVID-19 vaccine (*P* = 3.6987 × 10^–7^).

**Table 5:** KAP assessment response for each profession.

Item	Total (n = 395)	Physicians (n = 45)	Pharmacists (n = 73)	Nurses (n = 277)	*P* value
**Item.1: Knowledge**					
Do you know about the effectiveness of COVID-19 vaccines?					
Yes	316 (80%)	41 (91.1%)	61 (83.6%)	214 (77.3%)	0.069
No	79 (20%)	4 (8.9%)	12 (16.4%)	63 (22.7%)	
Do you know about the side effects of COVID-19 vaccines?					
Yes	329 (83.3%)	41 (91.1%)	61 (83.6%)	227 (81.9%)	0.31
No	66 (16.7%)	4 (8.9%)	12 (16.4%)	50 (18.1%)	
Can COVID-19 infection happen after taking the vaccine?					
Yes	272 (68.9%)	38 (84.4%)	68 (93.2%)	166 (59.9%)	**7.018 × 10**^**–9**^
No	36 (9.1%)	4 (8.9%)	0 (0%)	32 (11.6%)	
I don’t know	87 (22%)	3 (6.7%)	5 (6.8%)	79 (28.5%)	
Who should have been vaccinated?					
Those who have not yet been infected with COVID-19	28 (7.1%)	1 (2.2%)	2 (2.7%)	25 (9%)	0.31
People infected with COVID-19	8 (2%)	1 (2.2%)	2 (2.7%)	5 (1.8%)	
Elderly and patients with chronic diseases	70 (17.7%)	10 (22.2%)	10 (13.7%)	50 (18.1%)	
Everyone	289 (73.2%)	33 (73.3%)	59 (80.8%)	197 (71.1%)	
**Item.2: Attitude**					
The COVID-19 vaccines are essential for us					
Yes	267 (67.6%)	30 (66.7%)	55 (75.3%)	182 (65.7%)	0.07
No	44 (11.1%)	1 (2.2%)	6 (8.2%)	37 (13.4%)	
I don’t know	84 (21.3%)	14 (31.1%)	12 (16.4%)	58 (20.9%)	
I received at least one dose of a COVID-19 vaccine					
Yes	84 (21.3%)	21 (46.7%)	23 (31.5%)	40 (14.4%)	**3.6987 × 10**^**–7**^
No	311 (78.7%)	24 (53.3%)	50 (68.5%)	237 (85.6%)	
I will encourage my family/friends / relatives to get vaccinated					
Yes	248 (62.8%)	32 (71.1%)	46 (63%)	170 (61.4%)	**0.014**
No	58 (14.7%)	0 (0%)	16 (21.9%)	42 (15.2%)	
I don’t know	89 (22.5%)	13 (28.9%)	11 (15.1%)	65 (23.5%)	
**Item.3: Perception**					
Do you think that if everyone in society maintains the preventive measures, the COVID-19 pandemic can be eradicated without vaccination?					
Yes	98 (24.8%)	9 (20%)	28 (38.4%)	61 (22%)	**0.025**
No	204 (51.6%)	26 (57.8%)	35 (47.9%)	143 (51.6%)	
I don’t know	93 (23.5%)	10 (22.2%)	10 (13.7%)	73 (26.4%)	
What might convince you to take a COVID-19 vaccine OR convinced you if you already took it? (Choose all that apply)					
The people I know get the vaccine without issues	119 (30.1%)	14 (31.1%)	23 (31.5%)	82 (29.6%)	0.94
Getting the vaccine meant I would no longer have to wear a mask or social distance	60 (15.1%)	6 (13.3%)	12 (16.4%)	42 (15.2%)	0.901
Getting the vaccine can protect me from getting the infection	232 (58.7%)	33 (73.3%)	31 (42.5%)	168 (60.6%)	**0.002**
It is required for international travel	127 (32.2%)	19 (42.2%)	27 (37%)	81 (29.2%)	0.139
Nothing—I don’t believe vaccines are safe	47 (12%)	1 (2.2%)	11 (15.1%)	35 (12.6%)	0.088
Other reason	28 (7.1%)	5 (11.1%)	14 (19.2%)	9 (3.2%)	0.000008

Significant values are in bold.

**Figure 1:**
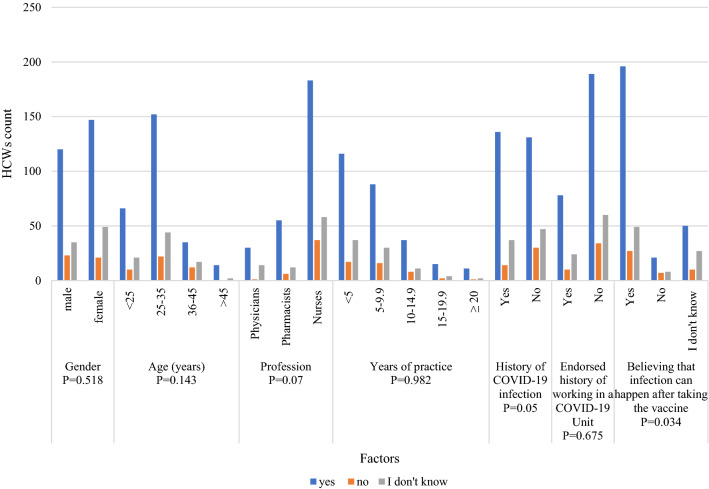
Factors affecting vaccination acceptance among HCWs.

**Figure 2:**
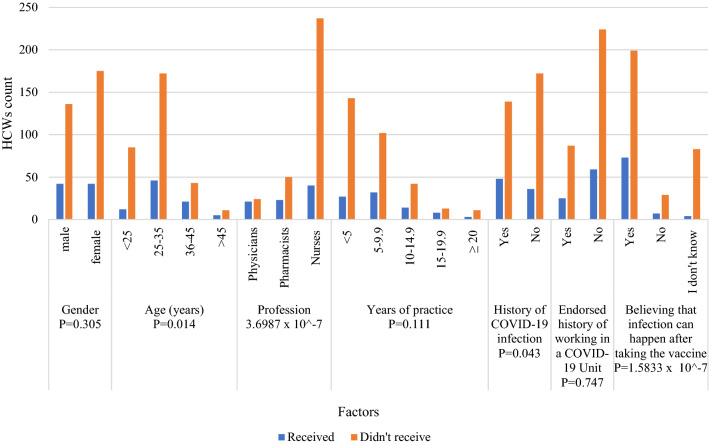
Factors affecting receiving at least one dose of a COVID-19 vaccine among HCWs.

**Figure 3:**
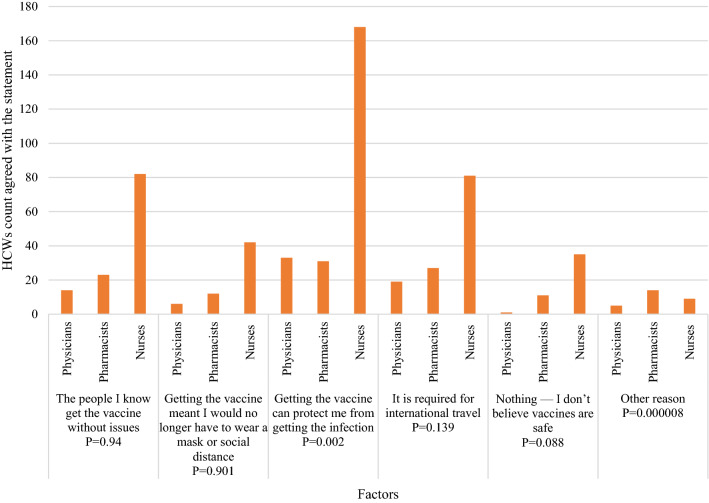
HCWs responses to “What might convince you to take a COVID-19 vaccine OR convinced you if you already took it? (Choose all that apply).

The vaccine acceptance was compared with several factors (Fig. 1). Nurses were significantly the most category through different professions who believe vaccines are essential for us (*P* = 0.07). Also, HCWs who agreed that vaccines are essential significantly believe that the infection can happen after taking the vaccine (*P* = 0.034). Furthermore, some factors significantly influenced the decision to receive at least one dose of COVID-19 vaccine, including the age between 25 and 35 years (*P* = 0.014), being a nurse (*P* = 3.6987 × 10^–7^), not having a history of COVID-19 infection (*P* = 0.043) and believing that the infection can happen after taking the vaccine (*P* = 7.018 × 10^–9^) (Fig. 2). In addition, pharmacists were the least HCWs convinced by the statement “Getting the vaccine can protect me from getting the infection” (*P* = 0.002) (Fig. 3).

## Discussion

Remarkable results from the current study have shown that more than half of HCWs (66%) who worked during the COVID-19 pandemic suffer from abnormal anxiety and depression symptoms. Those findings are consistent with published data during the pandemic inside and outside Egypt^4,5,12^. An online survey conducted during the first wave of the COVID-19 pandemic revealed that 40% of the participated HCWs had clinically significant emotional disorders. The results are also supported by a systematic review and meta-analysis that reported high prevalence rates for anxiety, depression, post-traumatic stress disorder, and distress 40%, 37%, 49%, and 37%, respectively^11^. High rates of fear, anxiety and abnormal psychological symptoms were expected and are known consequences of global pandemics^24^. During COVID-19, HCWs are predisposed to numerous factors that highly impact their mental health. Such factors include excessive workload, facing deaths daily, fear of getting the infection or transmitting it to their families, shortage of protective equipment, and lack of effective social support^6,25^. On top of that, the estimated mortality rate among oncology patients infected with COVID-19 was 20% due to weak immunity and postponing their cancer treatment regimens^26^. Hence, high anxiety and depression levels in our study could also be attributed to the fact that facing oncology patients is associated with additional distress^27^.

A highlighted finding embedded in the data is the significantly higher level of anxiety among nurses in comparison to physicians and pharmacists; (mild: nurses 99, physicians 25, pharmacists 24), (moderate: nurses 48, physicians 12, pharmacists 16), (severe: nurses 24, physicians 1, pharmacists 12), (*P* = 0.003). Nurses are the largest occupational staff in our facility, and they account for approximately 69% of the total healthcare task force. In addition, as per the World Health Organization (WHO) report, nurses and midwives comprise more than 50% of HCWs in many countries^28^. Moreover, they are considered the first and the direct line who interact consistently with patients^29^.

During COVID-19, severe degrees of psychological symptoms were reported in nursers rather than in other professions^5^. A cross-sectional study in the Philippines reported 37% (n = 325) of abnormal anxiety symptoms among frontline nurses. Interestingly, anxiety levels were lower in those with high levels of personal resilience and who received social and organizational support^30^. Another study found that nurses experienced higher levels of distress during previous SARS outbreaks. Suggested causes were fear for their health state, social isolation, and job workload^29^. Nurses account for the majority of the healthcare workforce worldwide. More importantly, HCWs who work long shifts, particularly nurses, are prone to sickness and absence^31,32^ In turn, it is essential to address the causes of their mental disorders and to provide efficient mental health support to them^32^.

Although our results indicated the lack of association between sociodemographics and the level of anxiety or depression, Alnazly et al. identified several factors positively correlated to psychological distress among HCWs. The determining factors were being male, older ages (above 40 years old), direct interaction with positive COVID-19 patients, and extended professional experience^33^. In contrast, Youssef et al. indicated that female HCWs and younger ages are more predisposed to adverse psychological symptoms^12^. We also reported another crucial finding: HCWs with a previous history of a positive COVID-19 infection expressed higher anxiety scores. Psychiatric comorbidities were previously reported consequence in COVID-19 survivors, as anxiety and depression likely happen in 42% and 31% of survivors, respectively^34^. In context, regardless of the differential factors that prone HCWs to significant psychological symptoms, they suffer from anxiety and depressive symptoms that need direct support and management.

Our findings point to the importance of psychological care and support that is urgent and required for all HCWs to decrease the levels of psychological distress. Several actions can be considered, such as emotional support programs provided by government and healthcare facilities^35,36^, encouragement of relaxation techniques such as yoga and meditation^37^, offering therapist’s visits when needed^38^. Additionally, shortening the working hours of^39^, consistently checking the staff who have burnout and need support (41), and conducting educational and training programs about mental health can also improve HCWs’ mental health status^40^. It is worth mentioning the successful prophylactic model “CREAT” implemented in Princess Margaret Cancer Centre, one of the world's largest cancer tertiary teaching hospitals. The program was implemented during the first wave of the COVID-19 pandemic (March 2020s) and targeted to provide support for oncology HCWs. It consisted of psychosocial coaches' support given to frontline HCWs to prevent burnout and emotional distress during the pandemic. The model included several interventions such as identifying HCWs' emotional needs, providing calming techniques, and providing online mental health support. The implementation of the such program led to a reduction in distress levels and an increase in team resilience^27^.

Adequate levels of knowledge, attitude, and perception toward COVID-19 vaccines were reported among HCWs. Our findings showed that the efficacy and safety of COVID-19 vaccines are well-recognized among HCWs (80%, 83.3%), respectively. Also, they have sufficient knowledge about COVID-19 vaccines, as reflected by the 68.9% who agreed that COVID-19 infection could happen after taking the vaccine and 73.2% who agreed that everyone should be vaccinated. Similar results were observed in a study conducted in three hospitals in Uganda in which 83.9% of the participants had good knowledge about COVID-19 vaccines^41^. Furthermore, HCWs in our facility reported that they do not believe that if everyone in society maintains the preventive measures, the COVID-19 pandemic can be eradicated without vaccination (*P* = 0.025). The knowledge level was expected to be high among HCWs giving reasons for their occupation and experience.

In different nations, vaccines still face doubt and rejection. The Centre of Disease Control and Prevention (CDC) reported that in the 2018–2019 flu season, only 45% of adults received a flu vaccination. In addition, about 35.8% of adults refuse to take the flu vaccine^18^. The vaccination acceptance rate in our study against COVID-19 was 67.6%. Closer rates were reported earlier in other studies on the general population (a global survey), HCWs, or both (71.5%, 64.9%, 60.6%), respectively^42–44^. Despite that, the percentage of HCWs who received at least one dose of a COVID-19 vaccine was only 21.3%. The difference between the acceptance percentage and the actual percentage of HCWs received the vaccine may be due to the limited vaccine availability at the survey time in Egypt.

Unexpectedly, nurses were the most profession who believed in COVID-19 vaccines, however, they were the least vaccinated group (P = 3.6987 × 10^–7^). An earlier study reported that medical doctors were more likely to accept the vaccine, but that was not statistically different among other professions^44^. Also, the educational level was insignificantly correlated with vaccine acceptance among Arabs^45^. Nurses in our institution lay within a wide age range, educational, and experience levels. Such diversity could contribute to the controversial result. Another factor significantly associated with a lower vaccination rate is believing that the infection can happen even after taking the vaccine and the lack of COVID-19 infection history (*P* = 1.5833 × 10–7), (*P* = 0.043), respectively. Such beliefs are actual even among HCWs, although all the available vaccines significantly protect from getting the infection and prevent hospitalization due to the infection^14^. Lastly, younger ages (< 25 and 25–35 years old) were the least vaccinated group (65%), which contradicts earlier findings reported by Elharake JA et al. in Saudi Arabia. In the later study, younger ages were more likely to accept the vaccines (49.2%), giving the reason for the extreme restrictions employed by Saudi authorities among their population aiming to control the pandemic^43^. However, fewer restrictions were employed in Egypt, which may lead younger HCWs to be less encouraged to take the vaccines.

Interestingly, 58.7% of our study sample agreed on taking the vaccine because it can protect them from getting the infection, 62.8% agreed on encouraging their relatives and friends to take the vaccine, while 12% refused to take it because they believe vaccines are not safe. In different countries, one of the major factors that potentially affect the decision to take the vaccines are side effects. A study conducted in the United States by Kreps et al. found that the vaccine acceptance rate is positively affected when the efficacy is higher, and the rate is reduced when the side effects are severe. Furthermore, a lower incidence of major side effects was associated with significantly higher acceptance rates^46^. Similarly, the most common reason for COVID-19 vaccine refusal among HCWs in Saudi Arabia was fear of side effects (59%)^43^. Those numbers indicate that HCWs can be encouraged to take the vaccines for their protection and control the pandemic for society. Also, raising the knowledge of the benefits of vaccines will likely increase the acceptance among HCWs. Concisely, better vaccination rates will be influenced by a better understating of the vaccine's benefits with facilitating the vaccination process.

We are aware that our research may have some limitations, given that the suggestive bias due to self-reporting of the assessment tools can result in some sources of error. Also, the nature of the cross-sectional study design as causality cannot be established as well as the single-centered model. Nevertheless, our study provided an overview of HCWs’ mental health in the middle of the pandemic. It also stratified the findings based on the profession that in turn, reported crucial results and summarized the factors that significantly impact the COVID-19 vaccination rate. Lastly, it offers an example of assessing and surveying HCWs in an organization and utilizing the results as a baseline for mental health educational programs.

## Conclusion

Our study was conducted during the COVID-19 pandemic, parallel with the emergence of COVID-19 vaccines. Approximately 66% of the study sample suffered from different anxiety and depressive symptoms. Increased anxiety levels were reported in nurses and those with a history of COVID-19 infection. In addition, despite the poor vaccination rate, sufficient knowledge, positive attitude, and perception toward COVID-19 vaccines were reported. Government and healthcare organizations must take serious interventions to decrease the mental load on HCWs. Also, to prioritize and facilitate the vaccination process for HCWs.

## Data Availability

The datasets used and/or analyzed during the current study are available from the corresponding author on reasonable request.
